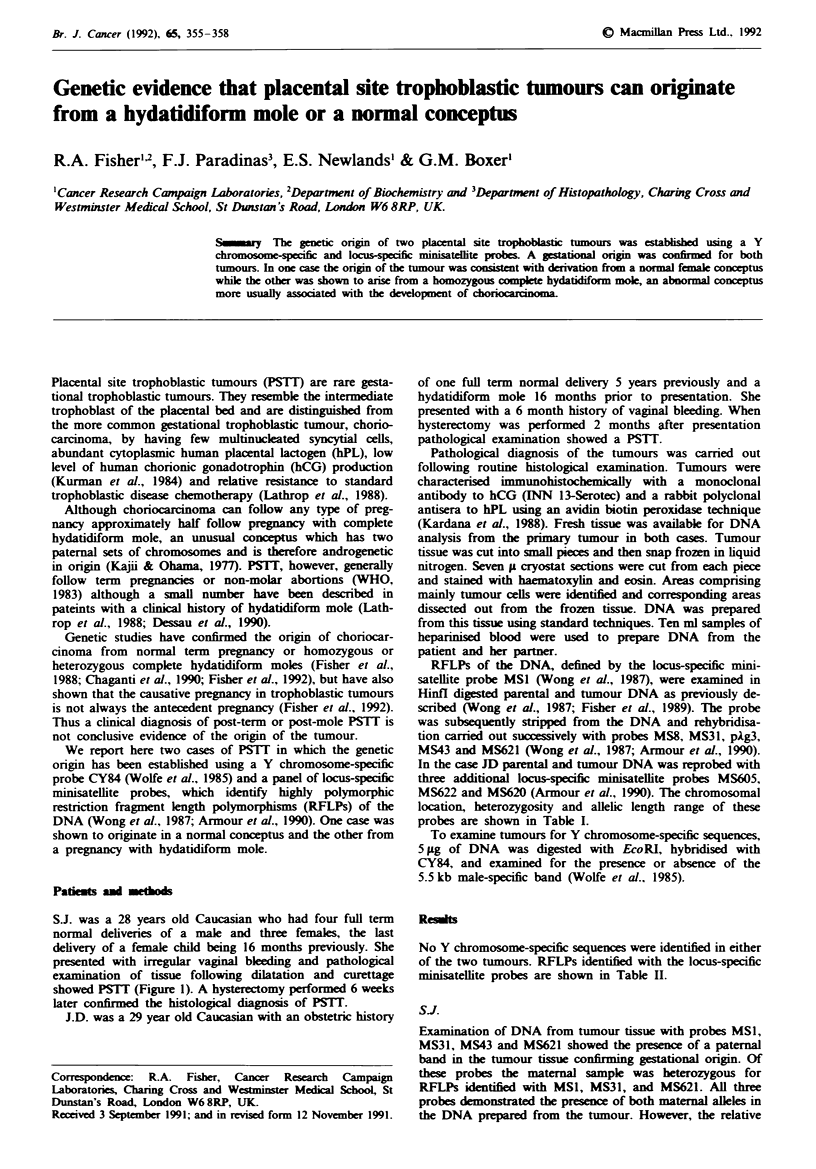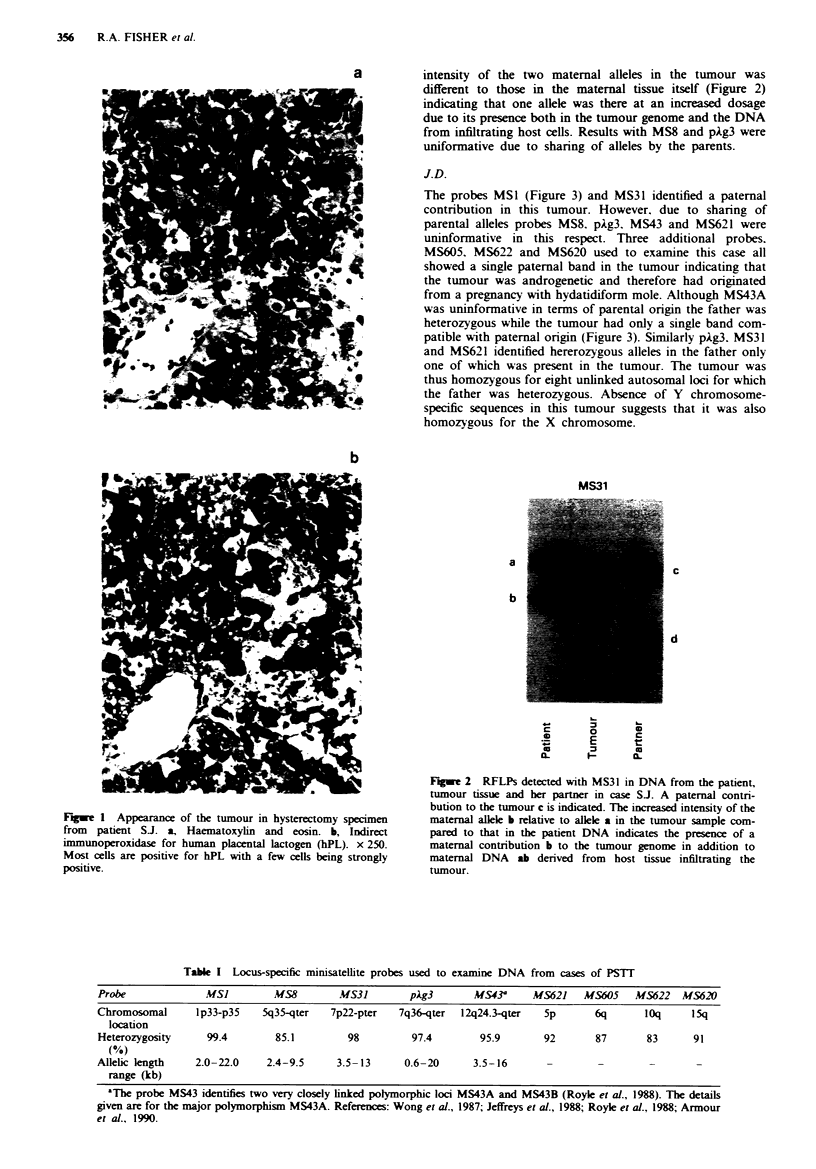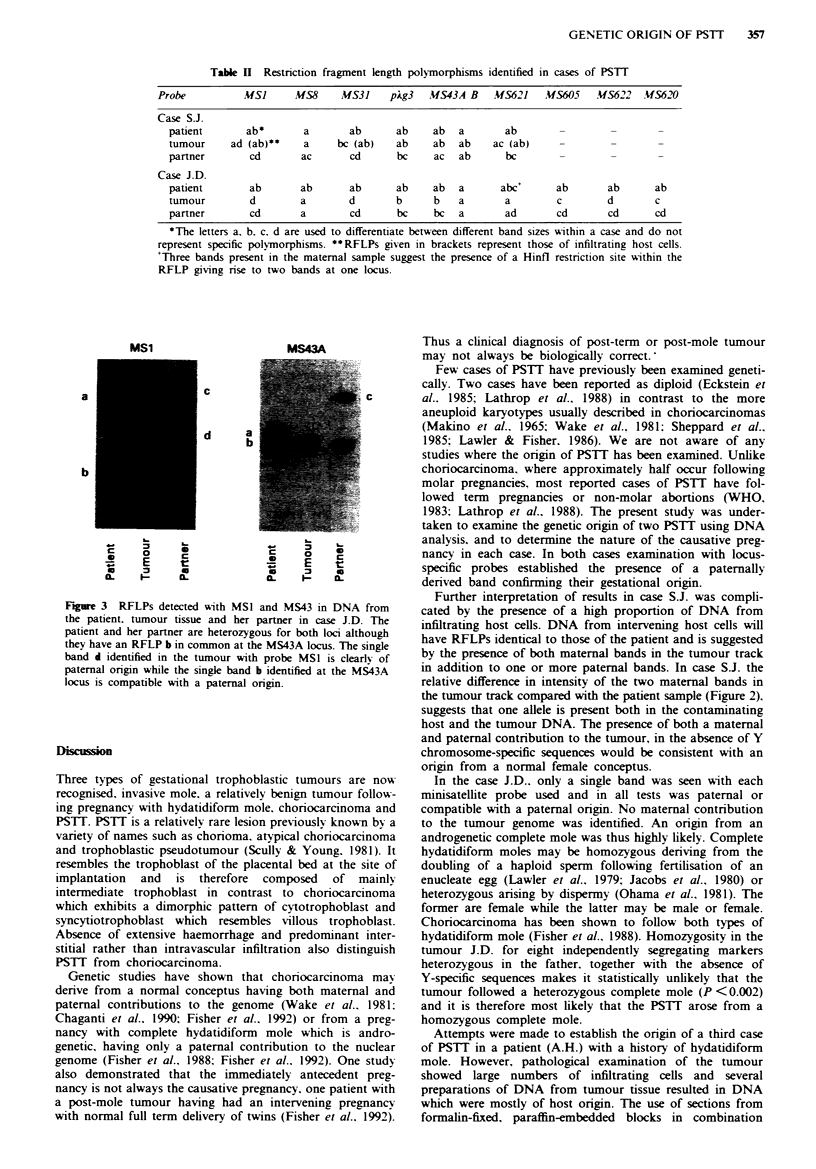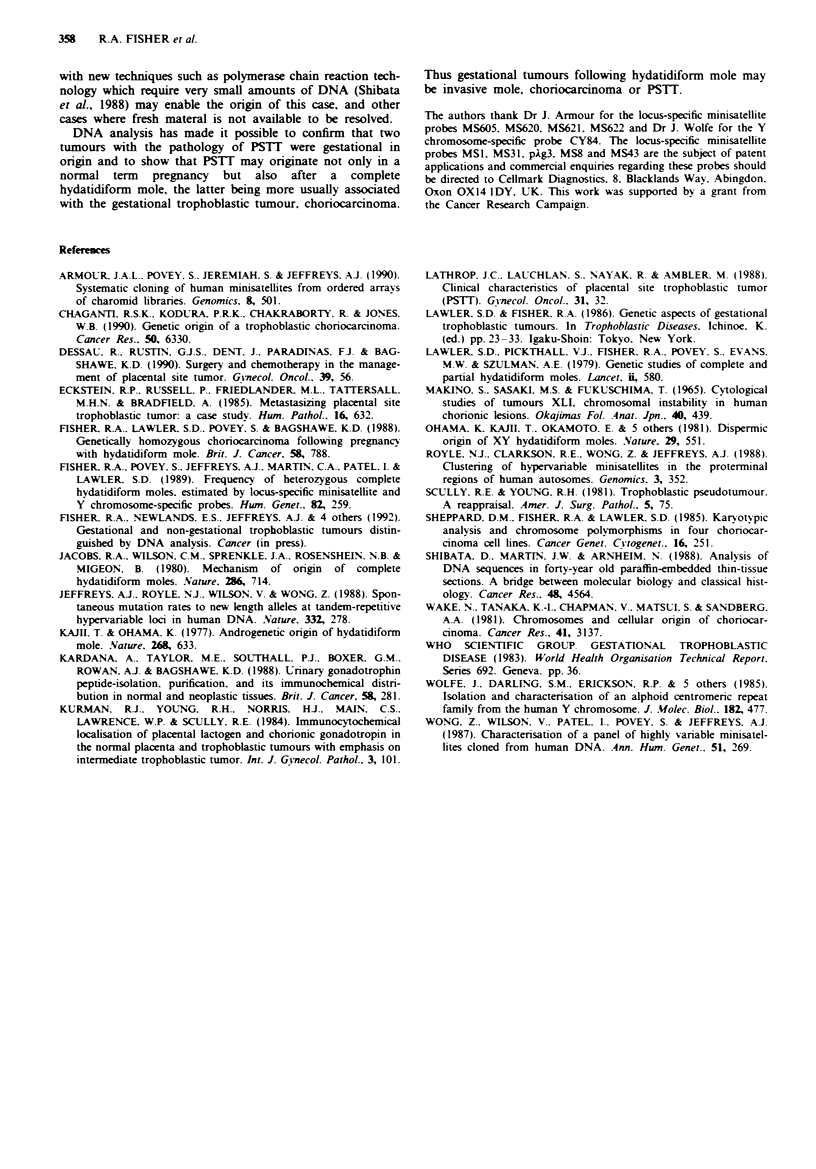# Genetic evidence that placental site trophoblastic tumours can originate from a hydatidiform mole or a normal conceptus.

**DOI:** 10.1038/bjc.1992.72

**Published:** 1992-03

**Authors:** R. A. Fisher, F. J. Paradinas, E. S. Newlands, G. M. Boxer

**Affiliations:** Cancer Research Campaign Laboratories, Charing Cross and Westminster Medical School, London, UK.

## Abstract

**Images:**


Br. J. Cancer (1992), 65, 355-358 © Macmillan Press Ltd., 1992

### Genetic evidence that placental site trophoblastic tumours can originate from a hydatidiform mole or a normal conceptus

R.A. Fisher[1,2], F.J. Paradinas[3], E.S. Newlands[1] & G.M. Boxer[1]

[1]*Cancer Research Campaign Laboratories,* [2]*Department of Biochemistry and* [3]*Department of Histopathology, Charing Cross and Westminster Medical School, St Dunstan's Road, London W6 8RP, UK.*

**Summary** The genetic origin of two placental site trophoblastic tumours was established using a Y chromosome-specific and locus-specific minisatellite probes. A gestational origin was confirmed for both tumours. In one case the origin of the tumour was consistent with derivation from a normal female conceptus while the other was shown to arise from a homozygous complete hydatidiform mole, an abnormal conceptus more usually associated with the development of choriocarcinoma.

Placental site trophoblastic tumours (PSTT) are rare gestational trophoblastic tumours. They resemble the intermediate trophoblast of the placental bed and are distinguished from the more common gestational trophoblastic tumour, choriocarcinoma, by having few multinucleated syncytial cells, abundant cytoplasmic human placental lactogen (hPL), low level of human chorionic gonadotrophin (hCG) production (Kurman *et al.*, 1984) and relative resistance to standard trophoblastic disease chemotherapy (Lathrop *et al.*, 1988).

Although choriocarcinoma can follow any type of pregnancy approximately half follow pregnancy with complete hydatidiform mole, an unusual conceptus which has two paternal sets of chromosomes and is therefore androgenetic in origin (Kajii & Ohama, 1977). PSTT, however, generally follow term pregnancies or non-molar abortions (WHO, 1983) although a small number have been described in pateints with a clinical history of hydatidiform mole (Lathrop *et al.*, 1988; Dessau *et al.*, 1990).

Genetic studies have confirmed the origin of choriocarcinoma from normal term pregnancy or homozygous or heterozygous complete hydatidiform moles (Fisher *et al.*, 1988; Chaganti *et al.*, 1990; Fisher *et al.*, 1992), but have also shown that the causative pregnancy in trophoblastic tumours is not always the antecedent pregnancy (Fisher *et al.*, 1992). Thus a clinical diagnosis of post-term or post-mole PSTT is not conclusive evidence of the origin of the tumour.

We report here two cases of PSTT in which the genetic origin has been established using a Y chromosome-specific probe CY84 (Wolfe *et al.*, 1985) and a panel of locus-specific minisatellite probes, which identify highly polymorphic restriction fragment length polymorphisms (RFLPs) of the DNA (Wong *et al.*, 1987; Armour *et al.*, 1990). One case was shown to originate in a normal conceptus and the other from a pregnancy with hydatidiform mole.

### Patients and methods

S.J. was a 28 years old Caucasian who had four full term normal deliveries of a male and three females, the last delivery of a female child being 16 months previously. She presented with irregular vaginal bleeding and pathological examination of tissue following dilatation and curettage showed PSTT (Figure 1). A hysterectomy performed 6 weeks later confirmed the histological diagnosis of PSTT.

J.D. was a 29 year old Caucasian with an obstetric history of one full term normal delivery 5 years previously and a hydatidiform mole 16 months prior to presentation. She presented with a 6 month history of vaginal bleeding. When hysterectomy was performed 2 months after presentation pathological examination showed a PSTT.

Pathological diagnosis of the tumours was carried out following routine histological examination. Tumours were characterised immunohistochemically with a monoclonal antibody to hCG (INN 13-Serotec) and a rabbit polyclonal antisera to hPL using an avidin biotin peroxidase technique (Kardana *et al.*, 1988). Fresh tissue was available for DNA analysis from the primary tumour in both cases. Tumour tissue was cut into small pieces and then snap frozen in liquid nitrogen. Seven μ cryostat sections were cut from each piece and stained with haematoxylin and eosin. Areas comprising mainly tumour cells were identified and corresponding areas dissected out from the frozen tissue. DNA was prepared from this tissue using standard techniques. Ten ml samples of heparinised blood were used to prepare DNA from the patient and her partner.

RFLPs of the DNA, defined by the locus-specific minisatellite probe MS1 (Wong *et al.*, 1987), were examined in HinfI digested parental and tumour DNA as previously described (Wong *et al.*, 1987; Fisher *et al.*, 1989). The probe was subsequently stripped from the DNA and rehybridisation carried out successively with probes MS8, MS31, pλg3, MS43 and MS621 (Wong *et al.*, 1987; Armour *et al.*, 1990). In the case JD parental and tumour DNA was reprobed with three additional locus-specific minisatellite probes MS605, MS622 and MS620 (Armour *et al.*, 1990). The chromosomal location, heterozygosity and allelic length range of these probes are shown in Table I.

To examine tumours for Y chromosome-specific sequences, 5 μg of DNA was digested with *Eco*RI, hybridised with CY84, and examined for the presence or absence of the 5.5 kb male-specific band (Wolfe *et al.*, 1985).

### Results

No Y chromosome-specific sequences were identified in either of the two tumours. RFLPs identified with the locus-specific minisatellite probes are shown in Table II.

### *S.J.*

Examination of DNA from tumour tissue with probes MS1, MS31, MS43 and MS621 showed the presence of a paternal band in the tumour tissue confirming gestational origin. Of these probes the maternal sample was heterozygous for RFLPs identified with MS1, MS31, and MS621. All three probes demonstrated the presence of both maternal alleles in the DNA prepared from the tumour. However, the relative

Correspondence: R.A. Fisher, Cancer Research Campaign Laboratories, Charing Cross and Westminster Medical School, St Dunstan's Road, London W6 8RP, UK.
Received 3 September 1991; and in revised form 12 November 1991.

**Figure 1** Appearance of the tumour in hysterectomy specimen from patient S.J. **a**, Haematoxylin and eosin. **b**, Indirect immunoperoxidase for human placental lactogen (hPL). × 250. Most cells are positive for hPL with a few cells being strongly positive.

intensity of the two maternal alleles in the tumour was different to those in the maternal tissue itself (Figure 2) indicating that one allele was there at an increased dosage due to its presence both in the tumour genome and the DNA from infiltrating host cells. Results with MS8 and pλg3 were uniformative due to sharing of alleles by the parents.

*J.D.*

The probes MS1 (Figure 3) and MS31 identified a paternal contribution in this tumour. However, due to sharing of parental alleles probes MS8, pλg3, MS43 and MS621 were uninformative in this respect. Three additional probes, MS605, MS622 and MS620 used to examine this case all showed a single paternal band in the tumour indicating that the tumour was androgenetic and therefore had originated from a pregnancy with hydatidiform mole. Although MS43A was uninformative in terms of parental origin the father was heterozygous while the tumour had only a single band compatible with paternal origin (Figure 3). Similarly pλg3, MS31 and MS621 identified hererozygous alleles in the father only one of which was present in the tumour. The tumour was thus homozygous for eight unlinked autosomal loci for which the father was heterozygous. Absence of Y chromosome-specific sequences in this tumour suggests that it was also homozygous for the X chromosome.

**Figure 2** RFLPs detected with MS31 in DNA from the patient, tumour tissue and her partner in case S.J. A paternal contribution to the tumour **c** is indicated. The increased intensity of the maternal allele **b** relative to allele **a** in the tumour sample compared to that in the patient DNA indicates the presence of a maternal contribution **b** to the tumour genome in addition to maternal DNA **ab** derived from host tissue infiltrating the tumour.

**Table I** Locus-specific minisatellite probes used to examine DNA from cases of PSTT

| *Probe* | *MS1* | *MS8* | *MS31* | *pλg3* | *MS43*[a] | *MS621* | *MS605* | *MS622* | *MS620* |
|---|---|---|---|---|---|---|---|---|---|
| Chromosomal location | 1p33-p35 | 5q35-qter | 7p22-pter | 7q36-qter | 12q24.3-qter | 5p | 6q | 10q | 15q |
| Heterozygosity (%) | 99.4 | 85.1 | 98 | 97.4 | 95.9 | 92 | 87 | 83 | 91 |
| Allelic length range (kb) | 2.0–22.0 | 2.4–9.5 | 3.5–13 | 0.6–20 | 3.5–16 | – | – | – | – |

[a]The probe MS43 identifies two very closely linked polymorphic loci MS43A and MS43B (Royle *et al.*, 1988). The details given are for the major polymorphism MS43A. References: Wong *et al.*, 1987; Jeffreys *et al.*, 1988; Royle *et al.*, 1988; Armour *et al.*, 1990.

**Table II** Restriction fragment length polymorphisms identified in cases of PSTT

| *Probe* | *MS1* | *MS8* | *MS31* | *pλg3* | *MS43A* | *B* | *MS621* | *MS605* | *MS622* | *MS620* |
|---|---|---|---|---|---|---|---|---|---|---|
| Case S.J. | | | | | | | | | | |
| patient | ab* | a | ab | ab | ab | a | ab | – | – | – |
| tumour | ad (ab)** | a | bc (ab) | ab | ab | ab | ac (ab) | – | – | – |
| partner | cd | ac | cd | bc | ac | ab | bc | – | – | – |
| Case J.D. | | | | | | | | | | |
| patient | ab | ab | ab | ab | ab | a | abc+ | ab | ab | ab |
| tumour | d | a | d | b | b | a | a | c | d | c |
| partner | cd | a | cd | bc | bc | a | ad | cd | cd | cd |

*The letters a, b, c, d are used to differentiate between different band sizes within a case and do not represent specific polymorphisms. **RFLPs given in brackets represent those of infiltrating host cells. +Three bands present in the maternal sample suggest the presence of a HinfI restriction site within the RFLP giving rise to two bands at one locus.

**Figure 3** RFLPs detected with MS1 and MS43 in DNA from the patient, tumour tissue and her partner in case J.D. The patient and her partner are heterozygous for both loci although they have an RFLP **b** in common at the MS43A locus. The single band **d** identified in the tumour with probe MS1 is clearly of paternal origin while the single band **b** identified at the MS43A locus is compatible with a paternal origin.

### Discussion

Three types of gestational trophoblastic tumours are now recognised, invasive mole, a relatively benign tumour following pregnancy with hydatidiform mole, choriocarcinoma and PSTT. PSTT is a relatively rare lesion previously known by a variety of names such as chorioma, atypical choriocarcinoma and trophoblastic pseudotumour (Scully & Young, 1981). It resembles the trophoblast of the placental bed at the site of implantation and is therefore composed of mainly intermediate trophoblast in contrast to choriocarcinoma which exhibits a dimorphic pattern of cytotrophoblast and syncytiotrophoblast which resembles villous trophoblast. Absence of extensive haemorrhage and predominant interstitial rather than intravascular infiltration also distinguish PSTT from choriocarcinoma.

Genetic studies have shown that choriocarcinoma may derive from a normal conceptus having both maternal and paternal contributions to the genome (Wake *et al.*, 1981; Chaganti *et al.*, 1990; Fisher *et al.*, 1992) or from a pregnancy with complete hydatidiform mole which is androgenetic, having only a paternal contribution to the nuclear genome (Fisher *et al.*, 1988; Fisher *et al.*, 1992). One study also demonstrated that the immediately antecedent pregnancy is not always the causative pregnancy, one patient with a post-mole tumour having had an intervening pregnancy with normal full term delivery of twins (Fisher *et al.*, 1992). Thus a clinical diagnosis of post-term or post-mole tumour may not always be biologically correct.

Few cases of PSTT have previously been examined genetically. Two cases have been reported as diploid (Eckstein *et al.*, 1985; Lathrop *et al.*, 1988) in contrast to the more aneuploid karyotypes usually described in choriocarcinomas (Makino *et al.*, 1965; Wake *et al.*, 1981; Sheppard *et al.*, 1985; Lawler & Fisher, 1986). We are not aware of any studies where the origin of PSTT has been examined. Unlike choriocarcinoma, where approximately half occur following molar pregnancies, most reported cases of PSTT have followed term pregnancies or non-molar abortions (WHO, 1983; Lathrop *et al.*, 1988). The present study was undertaken to examine the genetic origin of two PSTT using DNA analysis, and to determine the nature of the causative pregnancy in each case. In both cases examination with locus-specific probes established the presence of a paternally derived band confirming their gestational origin.

Further interpretation of results in case S.J. was complicated by the presence of a high proportion of DNA from infiltrating host cells. DNA from intervening host cells will have RFLPs identical to those of the patient and is suggested by the presence of both maternal bands in the tumour track in addition to one or more paternal bands. In case S.J. the relative difference in intensity of the two maternal bands in the tumour track compared with the patient sample (Figure 2), suggests that one allele is present both in the contaminating host and the tumour DNA. The presence of both a maternal and paternal contribution to the tumour, in the absence of Y chromosome-specific sequences would be consistent with an origin from a normal female conceptus.

In the case J.D., only a single band was seen with each minisatellite probe used and in all tests was paternal or compatible with a paternal origin. No maternal contribution to the tumour genome was identified. An origin from an androgenetic complete mole was thus highly likely. Complete hydatidiform moles may be homozygous deriving from the doubling of a haploid sperm following fertilisation of an enucleate egg (Lawler *et al.*, 1979; Jacobs *et al.*, 1980) or heterozygous arising by dispermy (Ohama *et al.*, 1981). The former are female while the latter may be male or female. Choriocarcinoma has been shown to follow both types of hydatidiform mole (Fisher *et al.*, 1988). Homozygosity in the tumour J.D. for eight independently segregating markers heterozygous in the father, together with the absence of Y-specific sequences makes it statistically unlikely that the tumour followed a heterozygous complete mole ($P < 0.002$) and it is therefore most likely that the PSTT arose from a homozygous complete mole.

Attempts were made to establish the origin of a third case of PSTT in a patient (A.H.) with a history of hydatidiform mole. However, pathological examination of the tumour showed large numbers of infiltrating cells and several preparations of DNA from tumour tissue resulted in DNA which were mostly of host origin. The use of sections from formalin-fixed, paraffin-embedded blocks in combination with new techniques such as polymerase chain reaction technology which require very small amounts of DNA (Shibata *et al.*, 1988) may enable the origin of this case, and other cases where fresh materal is not available to be resolved.

DNA analysis has made it possible to confirm that two tumours with the pathology of PSTT were gestational in origin and to show that PSTT may originate not only in a normal term pregnancy but also after a complete hydatidiform mole, the latter being more usually associated with the gestational trophoblastic tumour, choriocarcinoma. Thus gestational tumours following hydatidiform mole may be invasive mole, choriocarcinoma or PSTT.

The authors thank Dr J. Armour for the locus-specific minisatellite probes MS605, MS620, MS621, MS622 and Dr J. Wolfe for the Y chromosome-specific probe CY84. The locus-specific minisatellite probes MS1, MS31, pλg3, MS8 and MS43 are the subject of patent applications and commercial enquiries regarding these probes should be directed to Cellmark Diagnostics, 8, Blacklands Way, Abingdon, Oxon OX14 1DY, UK. This work was supported by a grant from the Cancer Research Campaign.

### References

ARMOUR, J.A.L., POVEY, S., JEREMIAH, S. & JEFFREYS, A.J. (1990). Systematic cloning of human minisatellites from ordered arrays of charomid libraries. *Genomics*, **8**, 501.

CHAGANTI, R.S.K., KODURA, P.R.K., CHAKRABORTY, R. & JONES, W.B. (1990). Genetic origin of a trophoblastic choriocarcinoma. *Cancer Res.*, **50**, 6330.

DESSAU, R., RUSTIN, G.J.S., DENT, J., PARADINAS, F.J. & BAGSHAWE, K.D. (1990). Surgery and chemotherapy in the management of placental site tumor. *Gynecol. Oncol.*, **39**, 56.

ECKSTEIN, R.P., RUSSELL, P., FRIEDLANDER, M.L., TATTERSALL, M.H.N. & BRADFIELD, A. (1985). Metastasizing placental site trophoblastic tumor: a case study. *Hum. Pathol.*, **16**, 632.

FISHER, R.A., LAWLER, S.D., POVEY, S. & BAGSHAWE, K.D. (1988). Genetically homozygous choriocarcinoma following pregnancy with hydatidiform mole. *Brit. J. Cancer*, **58**, 788.

FISHER, R.A., POVEY, S., JEFFREYS, A.J., MARTIN, C.A., PATEL, I. & LAWLER, S.D. (1989). Frequency of heterozygous complete hydatidiform moles, estimated by locus-specific minisatellite and Y chromosome-specific probes. *Hum. Genet.*, **82**, 259.

FISHER, R.A., NEWLANDS, E.S., JEFFREYS, A.J. & 4 others (1992). Gestational and non-gestational trophoblastic tumours distinguished by DNA analysis. *Cancer* (in press).

JACOBS, R.A., WILSON, C.M., SPRENKLE, J.A., ROSENSHEIN, N.B. & MIGEON, B. (1980). Mechanism of origin of complete hydatidiform moles. *Nature*, **286**, 714.

JEFFREYS, A.J., ROYLE, N.J., WILSON, V. & WONG, Z. (1988). Spontaneous mutation rates to new length alleles at tandem-repetitive hypervariable loci in human DNA. *Nature*, **332**, 278.

KAJII, T. & OHAMA, K. (1977). Androgenetic origin of hydatidiform mole. *Nature*, **268**, 633.

KARDANA, A., TAYLOR, M.E., SOUTHALL, P.J., BOXER, G.M., ROWAN, A.J. & BAGSHAWE, K.D. (1988). Urinary gonadotrophin peptide-isolation, purification, and its immunochemical distribution in normal and neoplastic tissues. *Brit. J. Cancer*, **58**, 281.

KURMAN, R.J., YOUNG, R.H., NORRIS, H.J., MAIN, C.S., LAWRENCE, W.P. & SCULLY, R.E. (1984). Immunocytochemical localisation of placental lactogen and chorionic gonadotropin in the normal placenta and trophoblastic tumours with emphasis on intermediate trophoblastic tumor. *Int. J. Gynecol. Pathol.*, **3**, 101.

LATHROP, J.C., LAUCHLAN, S., NAYAK, R. & AMBLER, M. (1988). Clinical characteristics of placental site trophoblastic tumor (PSTT). *Gynecol. Oncol.*, **31**, 32.

LAWLER, S.D. & FISHER, R.A. (1986). Genetic aspects of gestational trophoblastic tumours. In *Trophoblastic Diseases*, Ichinoe, K. (ed.) pp. 23–33. Igaku-Shoin: Tokyo, New York.

LAWLER, S.D., PICKTHALL, V.J., FISHER, R.A., POVEY, S., EVANS, M.W. & SZULMAN, A.E. (1979). Genetic studies of complete and partial hydatidiform moles. *Lancet*, **ii**, 580.

MAKINO, S., SASAKI, M.S. & FUKUSCHIMA, T. (1965). Cytological studies of tumours XLI, chromosomal instability in human chorionic lesions. *Okajimas Fol. Anat. Jpn.*, **40**, 439.

OHAMA, K, KAJII, T., OKAMOTO, E. & 5 others (1981). Dispermic origin of XY hydatidiform moles. *Nature*, **29**, 551.

ROYLE, N.J., CLARKSON, R.E., WONG, Z. & JEFFREYS, A.J. (1988). Clustering of hypervariable minisatellites in the proterminal regions of human autosomes. *Genomics*, **3**, 352.

SCULLY, R.E. & YOUNG, R.H. (1981). Trophoblastic pseudotumour. A reappraisal. *Amer. J. Surg. Pathol.*, **5**, 75.

SHEPPARD, D.M., FISHER, R.A. & LAWLER, S.D. (1985). Karyotypic analysis and chromosome polymorphisms in four choriocarcinoma cell lines. *Cancer Genet. Cytogenet.*, **16**, 251.

SHIBATA, D., MARTIN, J.W. & ARNHEIM, N. (1988). Analysis of DNA sequences in forty-year old paraffin-embedded thin-tissue sections. A bridge between molecular biology and classical histology. *Cancer Res.*, **48**, 4564.

WAKE, N., TANAKA, K.-I., CHAPMAN, V., MATSUI, S. & SANDBERG, A.A. (1981). Chromosomes and cellular origin of choriocarcinoma. *Cancer Res.*, **41**, 3137.

WHO SCIENTIFIC GROUP. GESTATIONAL TROPHOBLASTIC DISEASE (1983). *World Health Organisation Technical Report*, Series 692. Geneva. pp. 36.

WOLFE, J., DARLING, S.M., ERICKSON, R.P. & 5 others (1985). Isolation and characterisation of an alphoid centromeric repeat family from the human Y chromosome. *J. Molec. Biol.*, **182**, 477.

WONG, Z., WILSON, V., PATEL, I., POVEY, S. & JEFFREYS, A.J. (1987). Characterisation of a panel of highly variable minisatellites cloned from human DNA. *Ann. Hum. Genet.*, **51**, 269.